# Poor Correction Capacity of Preexisting Ankle Valgus Deformity after Total Knee Arthroplasty

**DOI:** 10.3390/jcm10163624

**Published:** 2021-08-17

**Authors:** Han-Ting Shih, Wei-Jen Liao, Kao-Chang Tu, Cheng-Hung Lee, Shih-Chieh Tang, Shun-Ping Wang

**Affiliations:** 1 Department of Orthopaedics, Taichung Veterans General Hospital, Taichung 40705, Taiwan; h901920@gmail.com (H.-T.S.); cavaliarjames@gmail.com (W.-J.L.); shark310751@gmail.com (K.-C.T.); 298f@vghtc.gov.tw (C.-H.L.); tom0857tom0857@gmail.com (S.-C.T.); 2 Department of Food Science and Technology, HungKuang University, Taichung 43302, Taiwan; 3 Sports Recreation and Health Management Continuing Studies-Bachelor’s Degree Completion Program, Tunghai University, Taichung 40704, Taiwan

**Keywords:** total knee arthroplasty, knee osteoarthritis, ankle malalignment, subtalar compensation, correctable

## Abstract

This study investigated the differences in ankle alignment changes after TKA in patients with varying preexisting ankle deformities. We retrospectively examined 90 knees with osteoarthritis and varus deformity in 78 patients who underwent TKA. Preoperative and postoperative radiographic parameters were analyzed. According to their preexisting ankle deformity, patients were assigned to the valgus or varus group. Overall, 14 (15.6%) cases were of preoperative valgus ankle deformity; the remainder were of preoperative varus ankle deformity. Hip–knee–ankle angle (HKA), tibial plafond–ground angle (PGA), and talus–ground angle (TGA) all exhibited significant correction in both groups; however, tibial plafond–talus angle (PTA) and superior space of ankle joint (SS) only changed in the varus group. The median PTA and SS significantly decreased from 1.2° to 0.3° (*p* < 0.001) and increased from 2.5 to 2.6 mm (*p* = 0.013), respectively. Notably, ∆PTA positively correlated with ∆HKA in the varus group (*r* = 0.247, *p* = 0.032) but not in the valgus group. Between-group differences in postoperative PTA (*p* < 0.001) and ∆PTA (*p* < 0.001) were significant. The degree of ankle alignment correction after TKA differed between patients with preexisting varus and valgus ankle deformities. TKA could not effectively correct the preexisting ankle valgus malalignment.

## 1. Introduction

Total knee arthroplasty (TKA) is considered the gold standard for treating end-stage knee osteoarthritis (OA). Studies have proved the effectiveness of TKA in alleviating pain and improving functional outcome [1,2]. However, in clinical scenarios, some patients complain of aggravation of ankle pain after surgery. Some of these patients have preexisting ankle OA [3,4,5,6]. Ankle pain following TKA is very troublesome and is associated with poorer clinical outcomes [3,5,7]. Therefore, to implement TKA in patients with both knee and ankle problems, one must consider possible postoperative changes in ankle alignment in the context of preventing ankle symptom exacerbation.

Severe knee OA is often comorbid with varus deformity of the knee. The aims of TKA are pain relief and reconstruction of the mechanical axis of the lower limb. TKA is performed to treat knees with OA and severe varus deformity, substantially correcting the angle of knee deformity and altering the alignment of the entire lower limb. Several radiological studies have asserted that changes in knee and lower limb alignment caused by TKA may also induce changes in the angle of the ankle joint [3,4,8,9,10,11,12].

When lower limb alignment is altered because of TKA, the alignment of the hindfoot, including the ankle and subtalar joints, is affected. To maintain balance and prevent overshift in the mechanical axis of the lower limb, the hindfoot uses a compensation mechanism. Regarding hindfoot compensation in the coronal plane, the ability of the subtalar joint is superior to that of the ankle joint. Several studies have reported changes in the angle of the subtalar joint after TKA [13,14,15,16,17].

Studies have proposed that limitations of subtalar compensation and preoperative ankle deformity are critical risk factors for post-TKA aggravation of ankle problems [17,18]. According to Lee et al., post-TKA ankle OA is more likely to occur in situations involving preoperative valgus deformity than in those involving preoperative varus deformity [3]. This may be because the compensation ability of the subtalar joint on the hindfoot differs depending on the preoperative ankle deformity. Numerous studies have confirmed that TKA leads to postoperative ankle alignment alterations [3,4,8,9,10,11,12]. However, regarding preexisting varus or valgus deformity of the ankle, few studies have explored whether TKA exerts different effects on ankle alignment correction. Furthermore, whether preoperative ankle deformities can be corrected after TKA remains a topic of debate [8,11,12].

To the best of our knowledge, this is the first investigation to focus on whether the degree of post-TKA ankle alignment alteration changes according to the types of preoperative ankle deformity of patients with varus OA knees. This study compared the extent to which ankle malalignment, with regard to preoperative valgus and varus deformity, can be corrected after TKA. We posited that preoperative varus ankle deformity is correctable after TKA but that preoperative valgus ankle deformity is uncorrectable.

## 2. Materials and Methods

### 2.1. Patient Enrollment

We conducted a retrospective search of patients receiving TKA between January 2010 and August 2015. The study protocol was approved by the institutional review board of our institution. The inclusion criteria were as follows. First, to reduce the potential effects of preoperative knee deformity on the results, we only included patients with varus knee deformities defined by a preoperative hip–knee–ankle angle (HKA) of >0°. Second, the patients must have undergone full-length standing anteroposterior radiography preoperatively and postoperatively. Third, the patients must have received a posterior-stabilized prosthesis. Patients who had received surgery on the ipsilateral lower extremity were excluded, as were patients with fracture of the ipsilateral lower extremity, abnormal congenital development, autoimmune diseases, infection, and incomplete medical records. All TKAs were performed by six experienced arthroplasty surgeons. During the operation, the posterior cruciate ligament was removed. Posterior stabilizing prostheses were used, the brand of which was determined according to the surgeon’s preference. A total of 90 knees in 78 patients were examined. Median patient age was 72.0 years (interquartile range [IQR]: 66.0 to 79.0). The operation was performed on the right leg, left leg, and both legs in 32, 34, and 12 of the patients, respectively. The median follow-up duration in this study was 14.9 (IQR: 9.2 to 31.3) months. The patients’ demographic data are shown in Table 1.

### 2.2. Study Grouping and Parameters of Knee and Ankle

**Figure 1:**
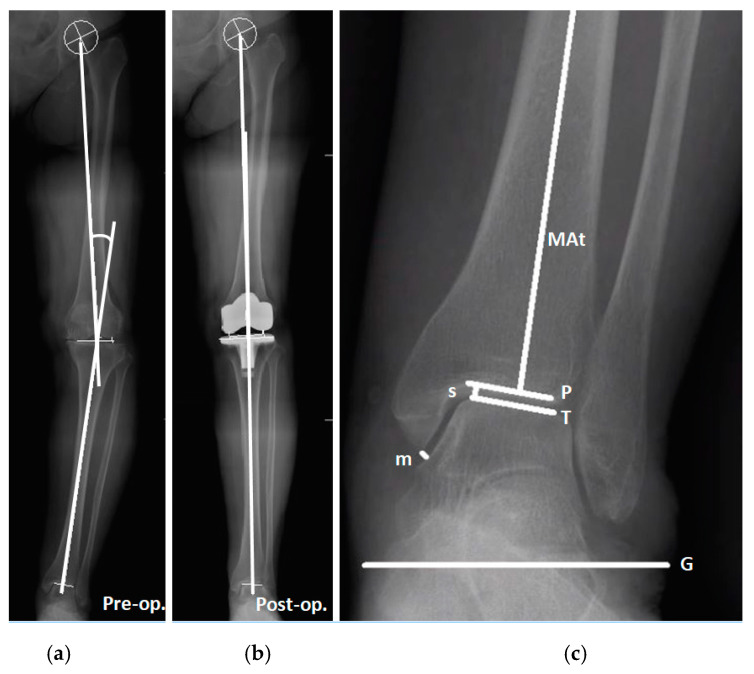
Preoperative and postoperative measurement of radiographic parameters for the knee and ankle in one patient. (**a**) Preoperative HKA measurement. Positive HKAs denote varus knee alignment, whereas negative HKAs denote valgus knee alignment. (**b**) Postoperative HKA measurement. (**c**) Ankle parameters: MAt line, mechanical axis of the tibia; P line, tibial plafond; T line, talar dome; G line, ground line. The LDTA is defined as the lateral angle of the MAt line and the P line. The PGA is defined as the angle between the P line and the G line. The TGA is defined as the angle between the T line and the G line. The PTA is defined as the angle between the P line and the T line. Positive PGA, TGA, and PTA values mean that the angle opens outward, whereas negative values mean that the angle opens inward. The MS and SS are indicated by m and s, respectively.

The patients’ preoperative and postoperative knee and ankle alignment angles were determined through full-length standing anteroposterior radiographs of the lower extremity, which involved the use of the picture archiving and communication system Ultraquery (Taiwan Electronic Data Processing, New Taipei City, Taiwan). The HKA was measured, as were six ankle parameters: the lateral distal tibial angle (LDTA), tibial plafond–ground angle (PGA), talus–ground angle (TGA), and tibial plafond–talus angle (PTA), as well as the medial space (MS) and superior space (SS) of the ankle joint (Figure 1). The preoperative–postoperative differences of these parameters were calculated and expressed as ∆HKA, ∆PGA, ∆TGA, ∆PTA, ∆MS, and ∆SS, respectively. All measurements were independently performed by two orthopedists, whose results were then averaged. According to the preoperative ankle deformity (with regard to the PTA of the ankle joint), the patients were assigned to the valgus group or the varus group, defined by a preoperative PTA of <0° and ≥0°, respectively.

### 2.3. Radiographic Measurements

Regarding the HKA measurement, the centers of the hip, knee, and ankle were identified on X-ray images taken before and after the operation. The angle between the line segments connecting the hip center and the knee center was measured, as was that connecting the center of the knee and ankle. The hip center was obtained using concentric Moose circles, whereas the ankle center was defined as the midpoint of the talar dome. The preoperative center of the knee was defined as the intersection of the tibial spine midline with that between the femoral condyles and the tibial tip (Figure 1a). After TKA, the center of the knee was defined as the intersection of the polyethylene inlay midline with that between the femoral condyles and the tibial tip (Figure 1b) [19]. An HKA of >0° indicates varus knee alignment, whereas an HKA of <0° indicates valgus knee alignment.

The LDTA was defined as the lateral angle of the mechanical axis of the tibia and the tibial plafond (Figure 1c) [20]. The PGA and TGA were defined as the angle between the tibial plafond and the ground and the angle between the talar dome and the ground, respectively. The PTA, also well known as the talar tilt angle, was defined as the angle between the tibial plafond and the talar dome (Figure 1c) [21]. Positive and negative PGA, TGA, and PTAs mean that the angle opens outward and inward, respectively. In other words, positive and negative PTAs are indicative of varus and valgus ankles, respectively.

The MS and SS (mm) were used to represent the ankle joint space (Figure 1c). The MS was defined as the vertical distance from the distal tip of the medial malleolus to the medial wall of the talus. The SS was defined as the shortest distance between the medial end of the talar dome and the tibial plafond.

### 2.4. Statistical Analysis

Analysis of between-group differences was conducted. Continuous variables were subjected to two-sided Mann–Whitney U tests, whereas categorical variables underwent a chi-square test or Fisher’s exact test. The Wilcoxon signed-rank test was performed to examine the preoperative–postoperative parameter differences. Correlation analysis was conducted using the Spearman correlation coefficient. All statistical analyses were performed using IBM SPSS Statistics for Windows, version 22 (IBM Corp., Armonk, NY, USA). The significance level was *p* < 0.05.

## 3. Results

As mentioned, 90 knees with OA and varus deformity were examined. The knees in the valgus (patients with preoperative PTA < 0°) and varus (patients with preoperative PTA ≥ 0°) groups numbered 14 and 76 (15.6% and 84.4%), respectively. The demographic data and preoperative radiographic parameters are listed in Table 1. No significant between-group differences were observed in age, sex, body mass index, or operation side. Regarding the preoperative radiographic parameters, the HKA, LDTA, PGA, TGA, and MS did not differ significantly between groups, but the PTA (median −1.3° vs. 1.2°; *p* < 0.001) and SS (median 3.0 vs. 2.5 mm; *p* = 0.021) did.

### 3.1. Radiographic Parameters of the Knee and Ankle Joint Change after Total Knee Arthroplasty in Patients with Different Preexisting Ankle Deformity

**Figure 2:**
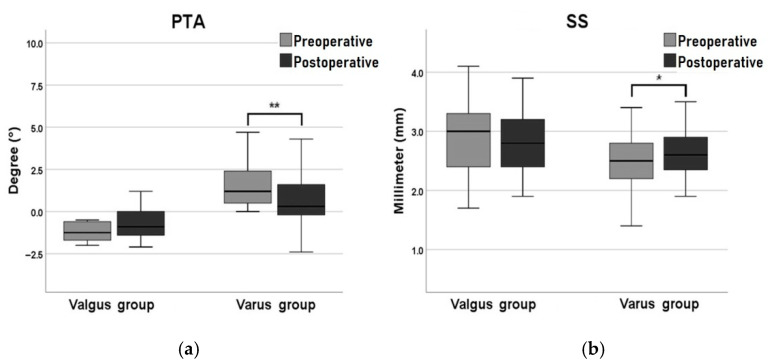
Post-TKA changes in (**a**) tibial plafond–talus angle (PTA) and (**b**) superior space (SS). ** *p* < 0.01, * *p* < 0.05.

After TKA, significant changes in the HKA, PGA, and TGA were observed in both groups. In the varus group, the median (IQR) HKA decreased significantly from 10.4° (6.9° to 14.3°) to 2.3° (0.9° to 4.8°; *p* < 0.001). The median (IQR) PGA and TGA significantly increased from −5.4° (−9.1° to −1.2°) to −1.1° (−4.5° to 3.2°); *p* < 0.001) and from −6.8° (−10.9° to −3.1°) to −1.7° (−5.5° to 2.6°; *p* < 0.001), respectively. In the valgus group, the median (IQR) HKA significantly decreased from 10.5° (3.6° to 12.3°) to 2.7° (0.5° to 5.0°; *p* = 0.001). The median (IQR) PGA and TGA significantly increased from −6.6° (−8.8° to −2.3°) to −1.6° (−3.9° to 1.8°; *p* = 0.017) and from −5.2° (−7.0° to −1.8°) to −0.1° (−3.1° to 2.6°; *p* = 0.008), respectively. Significant differences in the PTA and SS were observed only in the varus group. The median (IQR) PTA significantly decreased in the varus group from 1.2° (0.5° to 2.5°) to 0.3° (−0.2° to 1.6°; *p* < 0.001). In the valgus group, the median PTA changed from −1.3° to −0.9° (*p* = 0.197), a nonsignificant difference. The median (IQR) SS in the varus group (in millimeters) significantly increased from 2.5 (2.2 to 2.8) to 2.6 (2.3 to 2.9; *p* = 0.013), but the difference in the valgus group was nonsignificant (Table 2). In essence, only the preexisting ankle deformities in the varus group were corrected after TKA (Figure 2).

### 3.2. Correlation between Ankle Parameter Changes and HKA Changes in Patients with Different Preexisting Ankle Deformity

Regarding correlation analysis, in the varus group, ∆HKA was significantly negatively correlated with ∆PGA (*r* = −0.519, *p* < 0.001) and with ∆TGA (*r* = −0.615, *p* < 0.001). Furthermore, ∆HKA and ∆PTA (*r* = 0.247, *p* = 0.032) were significantly positively correlated. In the valgus group, ∆HKA was not significantly correlated with the changes in any of the ankle parameters (Table 3). Overall, for the patients with both varus knee and varus ankle deformities, the greater the degree of the knee deformity correction with regard to the TKA, the greater the degree of the ankle deformity correction.

### 3.3. Correlation between Ankle Parameter Changes and Preoperative HKA in Patients with Different Preexisting Ankle Deformity

To examine the association between the degree of varus deformity of the knee and the degree of ankle deformity correction in both varus and valgus groups, correlation analysis was conducted. Preoperative HKA significantly negatively correlated with ∆PTA in the varus group (*r* = −0.235, *p* = 0.041) but not in the valgus group (*r* = 0.029, *p* = 0.923) (Table 3). The extent of preoperative varus of the knee did partially affect the extent of the ankle correction.

### 3.4. Between Group Comparison of Radiographic Parameters in Patients with Different Preexisting Ankle Deformity

Difference analysis of the postoperative parameters and the preoperative–postoperative variations was conducted between groups. Significant parameter differences were not noted except for those of postoperative PTA and ∆PTA, which exhibited significant differences between groups. As shown in Table 4, the median (IQR) postoperative PTA differed significantly between groups: 0.3° (−0.2° to 1.6°) in the varus group and −0.9° (−1.4° to 0.0°; *p* < 0.001) in the valgus group. The median (IQR) ∆PTA in the varus and valgus groups was −0.7° (−1.6° to −0.2°) and 0.4° (−0.5° to 1.4°; *p* < 0.001), respectively. These results reveal that TKA exerted significantly different effects in the correction of varus and valgus ankle deformities.

## 4. Discussion

**Figure 3:**
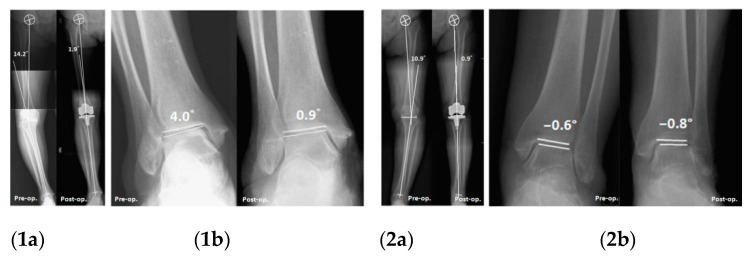
Ankle alignment changes after TKA in different preexisting ankle deformities. (1) Preexisting varus ankle deformity: (**1a**) HKA: 14.2 to 1.9°; (**1b**) PTA: 4.0 to 0.9°. (2) Preexisting valgus ankle deformity: (**2a**) HKA: 10.9 to 0.1°; (**2b**) PTA: −0.6 to −0.8°.

To the best of our knowledge, this study is the first to report significantly different effects of TKA on ankle alignment correction in patients with varying preexisting ankle deformities. Specifically, according to the results from the present study, varus ankle deformity was successfully corrected after TKA. The median (IQR) PTA decreased significantly from 1.2° (0.5° to 2.5°) to 0.3° (−0.2° to 1.6°; *p* < 0.001) in the varus group. Postoperatively, relatively neutral alignment was achieved by the majority of patients in the varus group (Figure 3). The extent of correction of the HKA was significantly positively correlated with that of the PTA (*r* = 0.247, *p* = 0.032). By contrast, changes in the ankle alignment of the valgus group did not exhibit clear correction; the deformity remained after TKA. To reiterate, preexisting ankle varus deformity was corrected to near neutral alignment after TKA, whereas preexisting ankle valgus deformity was not corrected.

Unlike primary OA of the hip or knee, primary ankle OA is rare. Most cases of ankle OA are classified as secondary OA, with the ankle as the affected part in 70 percent or more of cases of posttraumatic OA [22,23]. The correlation between ankle malalignment and ankle OA remains under debate. Abnormal ankle alignment causes tibial plafond–talus incongruence and changes in ankle biomechanics. Reductions in contact area increase the contact stress in the ankle joint, resulting in cartilage destruction that eventually leads to ankle OA [4,24,25,26]. Severe ankle malalignment can be managed and corrected in an expert surgeon’s hands. Many surgical procedures are indicated for end-stage ankle arthritis with wide coronal deformity, including arthrodesis and total ankle arthroplasty [27]. However, for those cases of mild malalignment of ankle without arthritic change, joint-sparing procedures may be more beneficial in the long term. In the present study, preoperative ankle varus deformity could be indirectly corrected after TKA, blocking the mechanism of ankle degradation. Moreover, the extent of knee alignment correction after TKA was positively correlated with the extent of ankle alignment correction in the varus group. For patients with substantial preexisting varus knee and ankle deformities, TKA can greatly correct knee deformity and, to a lesser extent, ankle deformity, resulting in fewer opportunities for ankle OA to develop. This may be one reason Lee et al. observed that preoperative valgus ankle deformity is more likely to cause ankle OA after TKA than is preoperative varus ankle deformity [3].

Ankle alignment can be measured through numerous methods. For example, some studies have used the angle between the ankle joint and the ground, including the PGA and TGA [4,8,9,11,12]. It is obvious that these two parameters change according to the alignment of the tibial bone corrected during TKA. Thus, that these parameters result in a position that is close to neutral after TKA is reasonable. However, the congruence between the tibial plafond and the talar dome, i.e., the PTA, and the degenerative process of the ankle are more closely linked. To the best of our knowledge, only few studies involving the determination of effects on the ankle from the radiographic evaluation of TKA have measured the PTA, the findings of which on the post-TKA effects on ankle alignment are inconsistent. Tonogai et al. reported changes in the PGA, TGA, and PTA after TKA [12], whereas Jeong et al. noted changes in only the TGA and PTA [11]. Gursu et al. indicated changes in the PGA but not the PTA [8]. In short, whether the PTA changes after TKA remains a topic of controversy. This inconsistency may be attributable to the lack of pre-TKA identification of different types of ankle malalignment. As mentioned, the PGA, TGA, and PTA all changed in the present varus group after TKA. Regarding the PTA changes after TKA, our result is consistent with that of Tonogai et al. but inconsistent with those of Jeong et al. and Gursu et al. However, we further observed that the PGA and TGA changed after TKA in the valgus group but that PTA did not. Significant between-group differences in the ∆PTA were detected. Therefore, we concluded that the correction (including the extent) of the PTA after TKA differed between the two groups. The PTA was corrected after TKA in the varus group but not in the valgus group.

The compensatory function of the hindfoot has been extensively studied. When severe deformities in lower limb joints (e.g., the ankle or knee) are surgically corrected, compensation by the subtalar joint sometimes occurs [13,14,15,16,17,28,29]. The fact that this phenomenon does not always occur suggests the presence of limiting factors in subtalar compensation [15,17,18,30]. Regarding the variation in preoperative ankle deformities, hindfoot compensation mechanisms may differ. According to Wang et al. and Colin et al., ankles with valgus deformity exhibit poorer compensation ability [15,18]. Through weight-bearing computed tomography (CT) of ankles with varus OA, Krahenbuhl et al. confirmed the greater occurrence of subtalar compensation with regard to varus ankle alignment. By contrast, no subtalar compensation was noted in cases of valgus ankle OA [17]. This could be linked to the natural range of motion of the subtalar joint [30]. Lee et al. suggested that patients with preoperative valgus ankle deformities could more easily develop ankle OA after TKA, possibly because the compensation ability of the subtalar joint in cases of preexisting valgus ankle deformities is weaker. The present study is the first to examine differences in two groups before and after TKA under the conditions of preoperative varus or valgus ankle deformities. The results indicate that the varus ankle deformities were correctable and well compensated, whereas the valgus ankle deformities were not. Therefore, before TKA is performed, in addition to assessing the alignment of the knee joint and the lower limbs, preexisting ankle deformities should be carefully assessed. Comprehensive preoperative planning for surgical alignment correction should be conducted in case problems with the knee joint are resolved but progressive ankle symptoms are exacerbated. However, progressive postoperative ankle symptoms ascribable to preexisting ankle deformities warrant further investigation.

This study has several limitations. First, this was a retrospective study; selection bias is a potential concern. Data loss and damage prevented us from effectively compiling data on functional outcomes. Furthermore, we did not take a time factor into account in this study. It is hard to control follow-up duration accurately due to the study’s retrospective nature. Several studies have reported the association between long-term exercise habit and osteoarthritis of the ankle [31,32]. To obtain more convincing conclusions, researchers can conduct prospective randomized studies that are well controlled. Second, the valgus group was smaller, potentially affecting statistical power; most of the cases were of varus deformity. However, the data conformed to distributions of general ankle deformity. In future investigations, case–control matching can be used to increase the sample size of participants with valgus ankle deformities, thereby increasing the reliability of statistical inferences. Finally, the radiographic parameters were all measured from full-length standing anteroposterior radiographs; no parameters were examined from the lateral view. Consequently, the measurements were less accurate when knee flexion contracture was involved. More precise measurements can be obtained by using three-dimensional weight-bearing CT. However, it is less clinically feasible because it increases patient exposure to radiation.

## 5. Conclusions

TKA produced different effects on ankle alignment depending on the nature of the preexisting ankle deformity (varus vs. valgus). TKA effectively corrected ankle alignment, including that with respect to the PGA, TGA, and PTA, in patients with both varus knee and varus ankle deformities. The greater the degree of the knee deformity correction, the greater the degree of the ankle deformity correction. TKA might effectively reduce the risk of ankle OA after the operation. In contrast, for patients with both varus knee and valgus ankle deformities, TKA exerted only weak benefits in ankle correction, and the likelihood of ankle OA development was high. Before TKA, comprehensive radiographic evaluation of ankle alignment is recommended. The preexisting ankle deformities should be addressed to avoid progressive ankle symptoms following TKA.

## Figures and Tables

**Table 1:** Demographic and baseline characteristics of cases.

	Total	Valgus Group (Preoperative PTA < 0°)	Varus Group (Preoperative PTA ≥ 0°)	*p*-Value ^a^
**Number** (%)	90	14 (15.6)	76 (84.4)	
**Age (years)**	72.0 [66.0, 79.0]	72.0 [68.8, 77.0]	72.0 [65.0, 79.0]	0.462
**Sex** **Male** (%) **Female** (%)	28 (31.1) 62 (68.9)	5 (35.7) 9 (64.3)	23 (30.3) 53 (69.7)	0.756
**BMI** (kg/m^2^)	27.7 [25.8, 30.9]	27.3 [24.5, 32.6]	27.7 [26.0, 30.8]	0.609
**Side** **Right** (%) **Left** (%)	44 (48.9) 46 (51.1)	6 (42.9) 8 (57.1)	38 (50.0) 38 (50.0)	0.623
**Preoperative parameters**				
**HKA** (°)	10.4 [6.4, 14.2]	10.5 [3.6, 12.3]	10.4 [6.9, 14.3]	0.379
**LDTA** (°)	88.1 [84.8, 89.9]	88.9 [86.0, 92.5]	88.0 [84.6, 89.5]	0.130
**PGA** (°)	−5.4 [−8.8, −1.5]	−6.6 [−8.8, −2.3]	−5.4 [−9.1, −1.2]	0.494
**TGA** (°)	−6.7 [−10.3, −3.0]	−5.2 [−7.0, −1.8]	−6.8 [−10.9, −3.1]	0.208
**PTA** (°)	0.9 [0.3, 2.3]	−1.3 [−1.7, −0.6]	1.2 [0.5, 2.5]	<0.001
**Medial space** (mm)	2.3 [1.8, 2.9]	2.3 [1.8, 2.8]	2.3 [1.8, 2.9]	0.978
**Superior space** (mm)	2.5 [2.2, 3.0]	3.0 [2.4, 3.3]	2.5 [2.2, 2.8]	0.021

Values are given as median with interquartile ranges [square brackets]. ^a^ Mann–Whitney U test applied for continuous variables, chi-square test or Fisher’s exact test for categorical variables. Significance level *p* < 0.05. BMI = body mass index, HKA = hip–knee–ankle angle, LDTA = lateral distal tibial angle, PGA = tibial plafond–ground angle, TGA = talus–ground angle, PTA = tibial plafond–talus angle.

**Table 2:** Preoperative and postoperative changes in radiographic parameters.

	Radiographic Parameters	Preoperative	Postoperative	*p*-Value ^a^
**Valgus group**	**HKA** (°)	10.5 [3.6, 12.3]	2.7 [0.5, 5.0]	0.001
	**PGA** (°)	−6.6 [−8.8, −2.3]	−1.6 [−3.9, 1.8]	0.017
	**TGA** (°)	−5.2 [−7.0, −1.8]	−0.1 [−3.1, 2.6]	0.008
	**PTA** (°)	−1.3 [−1.7, −0.6]	−0.9 [−1.4, 0.0]	0.197
	**Medial space** (mm)	2.3 [1.8, 2.8]	2.3 [1.7, 3.7]	0.937
	**Superior space** (mm)	3.0 [2.4, 3.3]	2.8 [2.4, 3.3]	0.537
**Varus group**	**HKA** (°)	10.4 [6.9, 14.3]	2.3 [0.9, 4.8]	<0.001
	**PGA** (°)	−5.4 [−9.1, −1.2]	−1.1 [−4.5, 3.2]	<0.001
	**TGA** (°)	−6.8 [−10.9, −3.1]	−1.7 [−5.5, 2.6]	<0.001
	**PTA** (°)	1.2 [0.5, 2.5]	0.3 [−0.2, 1.6]	<0.001
	**Medial space** (mm)	2.3 [1.8, 2.9]	2.5 [1.7, 3.1]	0.154
	**Superior space** (mm)	2.5 [2.2, 2.8]	2.6 [2.3, 2.9]	0.013

Values are given as median with interquartile ranges [square brackets]. ^a^ Wilcoxon signed-rank test. Significance level *p* < 0.05. HKA = hip–knee–ankle angle, PGA = tibial plafond–ground angle, TGA = talus–ground angle, PTA = tibial plafond–talus angle.

**Table 3:** Correlation between ankle parameter changes and HKA changes as well as preoperative HKA.

	Ankle Parameters	Correlation between Ankle Parameter Changes and ∆HKA		Correlation between Ankle Parameter Changes and Preoperative HKA	
		*r* ^a^	*p* ^a^	*r* ^a^	*p* ^a^
**Valgus group**	**∆PGA** (°)	−0.499	0.069	0.484	0.079
	**∆TGA** (°)	−0.526	0.053	0.543	0.045
	**∆PTA** (°)	−0.077	0.793	0.029	0.923
	**∆Medial space** (mm)	−0.115	0.695	0.341	0.233
	**∆Superior space** (mm)	0.156	0.593	−0.208	0.475
**Varus group**	**∆PGA** (°)	−0.519	<0.001	0.363	0.001
	**∆TGA** (°)	−0.615	<0.001	0.463	<0.001
	**∆PTA** (°)	0.247	0.032	−0.235	0.041
	**∆Medial space** (mm)	−0.116	0.32	0.073	0.531
	**∆Superior space** (mm)	−0.036	0.759	0.001	0.993

^a^ Spearman correlation analysis. Significance level *p* < 0.05. HKA = hip–knee–ankle angle, PGA = tibial plafond–ground angle, TGA = talus–ground angle, PTA = tibial plafond–talus angle.

**Table 4:** Between-group comparison of radiographic parameters.

	Ankle Parameters	Valgus Group	Varus Group	*p*-Value ^a^
**Postoperative**	**HKA** (°)	2.7 [0.5, 5.0]	2.3 [0.9, 4.8]	0.824
	**PGA** (°)	−1.6 [−3.9, 1.8]	−1.1 [−4.5, 3.2]	0.676
	**TGA** (°)	−0.1 [−3.1, 2.6]	−1.7 [−5.5, 2.6]	0.439
	**PTA** (°)	−0.9 [−1.4, 0.0]	0.3 [−0.2, 1.6]	<0.001
	**Medial space** (mm)	2.3 [1.7, 3.7]	2.5 [1.7, 3.1]	0.929
	**Superior space** (mm)	2.8 [2.4, 3.3]	2.6 [2.3, 2.9]	0.145
**Preoperative and Postoperative Differences**	**∆HKA** (°)	−6.4 [−11.0, −3.8]	−8.1 [−12.2, −4.8]	0.293
	**∆PGA** (°)	4.5 [2.0, 9.1]	4.9 [1.1, 7.4]	0.566
	**∆TGA** (°)	3.3 [2.2, 9.1]	5.4 [1.6, 8.0]	0.859
	**∆PTA** (°)	0.4 [−0.5, 1.4]	−0.7 [−1.6, −0.2]	<0.001
	**∆Medial space** (mm)	−0.1 [−0.2, 0.5]	0.0 [−0.3, 0.5]	0.499
	**∆Superior space** (mm)	0.0 [−0.2, 0.1]	0.1 [−0.1, 0.4]	0.100

Values are given as median with interquartile ranges [square brackets]. ^a^ Mann–Whitney U test. Significance level *p* < 0.05. HKA = hip–knee–ankle angle, PGA = tibial plafond–ground angle, TGA = talus–ground angle, PTA = tibial plafond–talus angle.

## Data Availability

All data are available upon reasonable request from the corresponding author.
